# The flavonoid nobiletin inhibits tumor growth and angiogenesis of ovarian cancers via the Akt pathway

**DOI:** 10.3892/ijo.2015.2946

**Published:** 2015-04-01

**Authors:** JIANCHU CHEN, ALLEN Y. CHEN, HAIZHI HUANG, XINGQIAN YE, WILLIAM D. ROLLYSON, HALEY E. PERRY, KATHLEEN C. BROWN, YON ROJANASAKUL, GARY O. RANKIN, PIYALI DASGUPTA, YI CHARLIE CHEN

**Affiliations:** 1 College of Biosystems Engineering and Food Science, Fuli Institute of Food Science, Zhejiang University, P.R. China; 2 College of Science, Technology and Mathematics, Alderson Broaddus University, Philippi, WV 26416, USA; 3 Department of Pharmaceutical Science, West Virginia University, Morgantown, WV 26506, USA; 4 Department of Pharmacology, Physiology and Toxicology, Joan C. Edwards School of Medicine, Marshall University, Huntington, WV 25755, USA

**Keywords:** nobiletin, ovarian cancer, angiogenesis, HIF-1α, vascular epithelial growth factor, Akt

## Abstract

Despite its importance, the death rate of ovarian cancer has remained unchanged over the past five decades, demanding an improvement in prevention and treatment of this malignancy. With no known carcinogens, targeted prevention is currently unavailable, and efforts in early detection of this malignancy by screening biomarkers have failed. The inhibition of angiogenesis, also known as angioprevention, is a promising strategy to limit the growth of solid tumors, including ovarian cancers. Nobiletin, a polymethoxy flavonoid compound isolated from the tiansheng plant, has been shown to inhibit the growth of multiple types of human cancers. However, there are no reports involving the effect on nobiletin on human ovarian cancer. The present report shows that nobiletin potently decreases the viability of ovarian cancer cells *in vitro*. However, nobiletin does not affect the viability of normal ovarian epithelial cells at <40 μM. The antitumor activity of nobiletin was also observed in athymic mouse models and in chicken chorioallantoic membrane (CAM) models. The anti-neoplastic activity of nobiletin was due to its ability to inhibit angiogenesis. We also studied the molecular mechanisms by which nobiletin suppresses angiogenesis. We observed that nobiletin inhibits secretion of the key angiogenesis mediators, Akt, HIF-1α, NF-κB and vascular epithelial growth factor (VEGF) by ovarian cancer cells. Transient transfection experiments showed that nobiletin inhibits production of HIF-1α by downregulation of Akt. Such decreased levels of HIF-1α were responsible for nobiletin-induced suppression of VEGF. Our data suggest that nobiletin may be a promising anti-angiogenic agent relevant for therapy of ovarian cancers.

## Introduction

Ovarian cancer is one of the most serious malignancies for women in the world, ranking as the fifth leading cause of cancer-related deaths (1) and the death rate of ovarian cancer has not seen any remarkable changes for over five decades in the USA (2). Due to a lack of effective biomarkers for screening (3,4), nearly 60–70% of ovarian cancers are diagnosed at advanced stages (5), with a poor prognosis of ~30% for a 5-year survival rate (6). These facts emphasize the need for novel therapies to prevent and treat ovarian cancer. Several lines of evidence indicate that nutritional compounds display potent anticancer activity in many human cancers (5). The identification of nutritional agents that can suppress the growth and progression of ovarian cancer could lead to new treatment modalities and improved patient outcomes for this lethal malignancy.

Recent studies have focused on the anticancer activity of flavonoids isolated from plants and animals. Flavonoids are natural polyphenols present in a wide variety of fruits and vegetables (7). Some flavonoids, such as apigenin, genistein and catechin, have been shown to inhibit the growth of ovarian, breast, colon, prostate and leukemia cancer cells (8–17). Nobiletin (5,6,7,8,30,40-hexamethoxyflavone) is a polymethoxyflavonoid found in citrus fruits such as *Citrus depressa* and *Citrus reticulate* (18). Previous mammalian *in vivo* studies show that nobiletin can suppress inflammation-associated tumorigenesis aberrant cell proliferation and colon carcinogenesis (19–21). Nobiletin has been also shown to suppress angiogenesis *in vitro* in human umbilical vein endothelial cells. The anti-angiogenic activity of nobiletin has been shown in chicken chorioallantoic membranes and in zebrafish models (22,23). It has been revealed that nobiletin exhibited a cell differentiation-modulating activity (24,25) and inhibited the phosphorylation of MEK (26,27). Nobiletin is a decreases metastasis of human fibrosarcoma HT-1080 cells and gastric cancers (27). The mitogen-activated protein kinase (MAPK) signaling pathway is a key regulator of cell proliferation, survival and differentiation. The MAPK pathway is constitutively activated in ovarian cancers via gain of function mutations in Ras or Raf. In addition, mutations in PI-3 kinase pathway have been implicated in the progression of ovarian cancers. The hyperactivation of the MAP kinase pathway facilitates the neoplastic transformation of ovarian tumors. Selective MEK1 inhibitors have been shown to suppress the growth of estrogen-responsive ovarian cancers. Since nobiletin also functions as a MEK1 inhibitor we conjectured that perhaps it could suppress the growth of human ovarian cancers. The growth-inhibitory activity of nobiletin has yet to be studied in ovarian cancer. Our report fills this void of knowledge and describes the anti-neoplastic activity of nobiletin in human ovarian cancer. In this report, we show that nobiletin decreases the viability of the human ovarian cancer cell lines OVCAR-3 and CP-70. The growth-inhibitory effects of nobiletin were observed at concentration as low as 5 μM. Nobiletin had no effect on the viability of normal ovarian epithelial cells at <40 μM. Therefore, it displayed a strong selectivity for human ovarian cancer cells over normal ovarian cells. Nobiletin potently decreased the growth rate of human ovarian tumors xenografted in athymic mouse models and chicken CAM models. The anticancer activity of nobiletin was correlated with its anti-angiogenic and anti-apoptosis activity in ovarian cancers. We also analyzed the signaling pathways underlying the anti-angiogenic activity of nobiletin. The anti-angiogenic activity of nobiletin was correlated with decreased levels of Akt, HIF-1α, NF-κB and vascular epithelial growth factor (VEGF) in ovarian cancer cells. Taken together, our data suggest that nobiletin may have applications in the therapy of human ovarian cancer.

## Materials and methods

### Ethics statement

Male four-week-old athymic mice were obtained from Charles River Laboratories and acclimatized for one week. They were housed in autoclaved cages with *ad libitum* access to food and water in HEPA-filtered racks and closely monitored by animal facility staff. All procedures involving nude mice were conducted according to the Animal Care and Use guidelines in a facility accredited by the Association for Assessment and Accreditation of Laboratory Animal Care (AAALAC) International and were approved by the Institutional Animal Care and Use Committee (IACUC) of Joan C. Edwards School of Medicine, Marshall University (protocol no. 560).

### Reagents, antibodies and constructs

**Figure 1:**
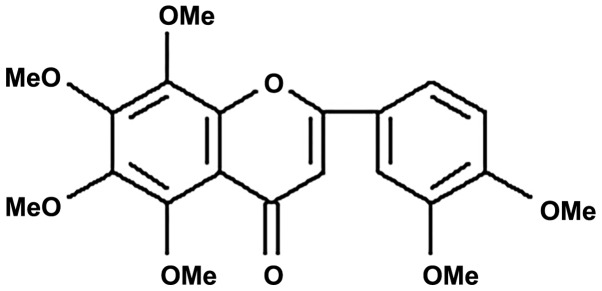
Structure of nobiletin.

Nobiletin was prepared from a polymethoxyflavonoid mixture, which was provided by Zhejiang Quzhou Tiansheng Plant Extraction Co. Ltd. in China, containing ~60% nobiletin and tangeretin. The polymethoxyflavonoid mixture was dissolved in methanol-dimethyl sulfoxide (1:1), its concentration was 50 mg/ml, then chromatographed with high-performance liquid chromatography (HPLC) (Waters) eluted with methanol-H_2_O (70:30) in 8 ml/min at room temperature, separated into two fractions, collected individually, evaporated, obtained fraction I and fraction II. Fraction I was identified as nobiletin (Fig. 1A) by HPLC-MS, UV-vis chromatography and comparing peak time with that of nobiletin sample from Sigma and previous reports (data not shown). Its purity was >98%. Monoclonal antibodies against HIF-1α, NF-κB (p50), PTEN, c-Myc, GAPDH, p-AKT, total AKT, p-mTOR and total mTOR were purchased from Santa Cruz Biotechnology (Santa Cruz, CA, USA). The secondary antibodies of anti-rabbit and anti-mouse were purchased from Thermo Scientific (Pierce, Rockford, IL, USA). The Hif -1α and mAkt plasmid constructs were obtained from Addgene (www.addgene.org) (28).

### Cell culture and treatment

Human ovarian cancer cell lines, OVCAR-3 and A2780/CP70, were provided by Dr B. Jiang, Department of Microbiology, Immunology, and Cell Biology, West Virginia University. IOSE-364, normal ovarian surface epithelial cells from healthy women, but immortalized with SV40 T/t, were courtesy of Dr N. Auersperg at University of British Columbia, Canada. All cells were maintained in RPMI-1640 medium supplemented with 100 U/ml penicillin, 100 μg/ml streptomycin and 10% US-qualified fetal bovine serum (Invitrogen, Grand Island, NY, USA) in a humidified incubator with 5% CO_2_ at 37°C. Nobiletin was dissolved in dimethyl sulfoxide (DMSO) to make stock solutions of 100 mM and equal amount of DMSO was included in controls for every experiment.

### Cell proliferation

The effect of nobiletin on the viability of ovarian cancer cells (OVCAR-3 and A2780/CP70) colorimetrically determined with a CellTiter 96 Aqueous One Solution Cell Proliferation Assay kit from Promega (Madison, WI, USA). The cells were seeded into 96-well plates at a density of 5×10^3^/well and incubated for 24 h at 37°C. Subsequently, the cells were treated with vehicle or varying concentrations of nobiletin for another 24 h at 37°C. After 24 h, the medium was removed and cell viability was measured according to the manufacturer’s instructions. Each sample was measured in triplicate. Cell viability was expressed as percentage of control from three independent experiments.

### Apoptosis assay

The apoptotic effects of nobiletin on ovarian cancer cells were determined by FITC Annexin V Apoptosis Detection Kit I from BD Biosciences. Cells were washed with cold PBS twice and then were resuspended in binding buffer at a concentration of 1×10^6^/ml. An aliquot of 100 μl of the cell solution (1×10^5^ cells) was transferred to a 5-ml tissue culture tube. Subsequently, 5 μl of FITC Annexin V and 5 μl propidium iodide (PI) was added to the cells. The cells were gently vortexed and incubated for 15 min at room temperature in the dark. The next step involved the addition of 400 μl of 1X binding buffer to each tube. The samples were analyzed by flow cytometry (Cytomic FC 500MCL) within 1 h. Three independent experiments were assayed. Data represent mean ± SE from 3 independent experiments.

### ELISA for VEGF

The levels of VEGF in cell culture supernatants were analyzed by a Quantikine Human VEGF Immunoassay kit (R&D Systems, Minneapolis, MN, USA). Cells (1×10^4^/well) were seeded into 96-well plates and incubated overnight. Subsequently, the cells were treated with nobiletin for 16 h in serum-free medium. Culture supernatants were collected and spun down at 10,000 g at 4°C. The supernatant was collected and stored at −70°C. The amounts of VEGF were measured following the manufacturer’s instructions, and normalized to cell numbers for each treatment. A total of three independent experiments, each in triplicates, were assayed, and the mean VEGF protein level from each triplicate was used for statistical analysis.

### Western blot analysis

Ovarian cancer cells (10^6^) were seeded in 60-mm dishes and incubated for 16 h before treated with nobiletin for 24 h. The cells were washed with PBS, lysed in 100 μl mammalian protein extraction reagent including 1 l halt protease, 1 μl phosphatase inhibitor and 2 μl EDTA (M-PER, Pierce), according to the manufacturer’s instructions. Total protein levels were assayed with a BCA Protein Assay kit (Pierce). Forty microgram of protein lysates was separated by 10% SDS-PAGE and transferred into nitrocellulose membrane with a Mini-Protean 3 system (Bio-Rad Laboratories, Hercules, CA, USA). The membranes were blocked in 5% milk in PBS containing 0.1% Tween-20 for 1 h at room temperature. The membranes were incubated with the appropriate dilutions of the primary antibodies and secondary antibodies. The signal obtained in the western blot experiments was detected by the SuperSignal West Dura Extended Duration Substrate (Pierce Biotechnologies). Protein bands were quantitated with NIH ImageJ software, normalized by corresponding GAPDH or total AKT, total mTOR bands, and expressed as percentages of control. A total of three independent experiments were carried out for statistical analysis.

### Transient transfection and reporter assay

Transient transfection and reporter assay were modified from our published report (28,29). Ovarian cancer cells were seeded in 96-well plate at 10,000 cells/well and incubated overnight. For transfection with HIF-1α/mAkt plasmids, cells were then transfected with 0.05 μg VEGF luciferase reporter, 0–0.25 μg HIF-1α/mAkt or SR-α (as vehicle) plasmids by 0.6 μl jetPRIME reagent (VWR) for 4 h and removed the medium. Followed by 16-h treatment with 0- or 40-μM nobiletin. The cells were harvested and analyzed for luciferase activities with ONE-Glo Luciferase Assay system (Promega) and detected by Lumat LB9507 (Berthold Technologies). Total protein levels with a BCA Protein Assay kit (Pierce), and the activities of VEGF reporter were normalized by corresponding total protein levels for statistical analysis. The experiments were conducted three times.

### Chicken embryo chorioallantoic membrane (CAM) assay

The A2780 cells at grown to 70% confluence, were harvested, washed with PBS and re-suspended in serum-free medium. Aliquots of the cells (0.1 ml, 2×10^7^/ml) were mixed with 0.1 ml of Matrigel (BD Bioscience, San Jose, CA, USA), supplemented with 0 or 20 μM nobiletin, pre-gelled on an autoclaved silicone mat for 30–40 min, and implanted onto the CAM of 9-day-old chicken embryo. Chicken embryos were incubated for 4–5 days, photographed for Matrigel implant, and counted for branching blood vessels. Angiogenesis was evaluated by normalizing number of branching vessels to that of control CAM. A total of 10 eggs were assayed for each group.

### Antitumor studies in athymic mice

Twenty-four week-old male nude mice were obtained from Charles River Laboratories and acclimatized for one week. They were housed in autoclaved cages with *ad libitum* access to food and water in HEPA-filtered racks and closely monitored by animal facility staff. All procedures were conducted according to the Animal Care and Use guidelines in a facility accredited by the Association for Assessment and Accreditation of Laboratory Animal Care (AAALAC) International and were approved by the Institutional Animal Care and Use Committee (IACUC) of Joan C. Edwards School of Medicine, Marshall University (protocol no. 560).

CP70 cells were harvested and re-suspended in a 1:1 (v/v) solution of serum-free media and Matrigel matrix (BD Biosciences). Two million cells in 100 μl were injected subcutaneously between the scapulae of each mouse (30). After the tumors reached 100 mm^3^, the mice were randomized and divided into two groups comprising of ten mice each. The treatment group (N=10) was fed AIN-76A diet with 5% lipid level containing 100 mg nobiletin/kg food. The control group (N=10) was fed AIN76A diet containing vehicle. Mice were weighed once per week. Their food consumption was monitored daily. Tumor volumes were calculated as (l × w^2^)/2 (31,32).

### Statistical analysis

Results are expressed as mean ± standard error of mean (SEM) using Microsoft Excel (Windows 8). Statistical assessment was carried out with the program system of SPSS (Version 16.0 for Windows). The results were analyzed using one-way analysis of variance (ANOVA) and *post hoc* test (2-sided Dunnett’s test) to test both overall differences and specific differences between each treatment and control. A P-value of <0.05 was considered statistically significant.

## Results

### Effect of nobiletin on ovarian cancer cell viability

**Figure 2:**
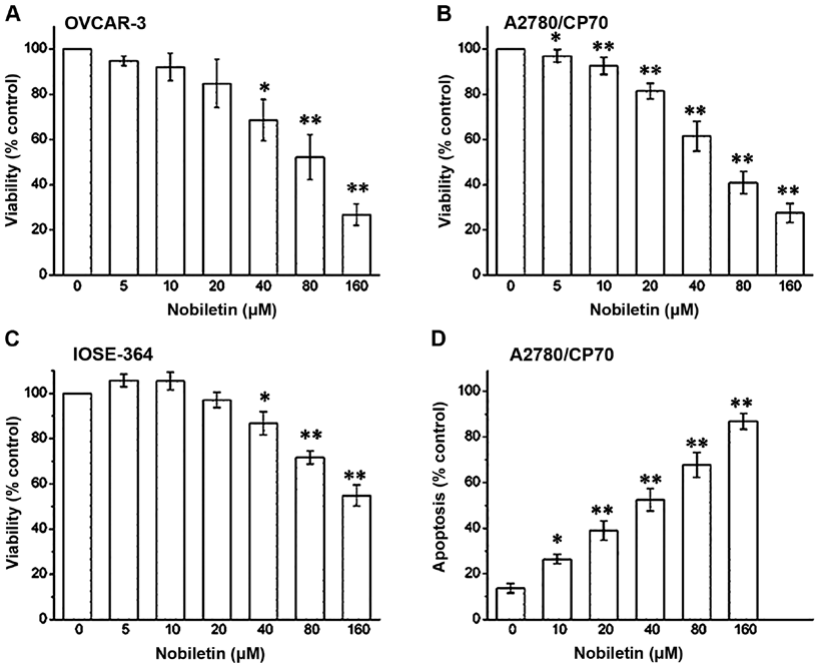
Nobeletin decreased the viability and induced apoptosis of human ovarian cancer cells in a concentration-dependent manner. (A) Cell viability assays showed that the treatment of OVCAR-3 cells with varying concentrations of nobiletin caused a decrease in cell viability. (B) The cell viability assay was repeated in A2780/CP70 cells. (C) Cell viability results were obtained in IOSE-364 cells. (D) Apoptosis assay showed that nobiletin induced apoptosis of A2780/CP70 ovarian cancer cells. Data represent mean ± SE from 3 independent experiment. ^*^P<0.05 as compared to control. ^**^P<0.01 as compared to control.

The treatment of OVCAR-3 and CP70 ovarian cancer cells with nobiletin caused a concentration-dependent decrease in cell viability over 24 h. Beginning at a concentration of 5 μM nobiletin, OVCAR-3 cell viability consistently decreased from 95±1 to 28±4% at a concentration of 160 μM nobiletin (P<0.01) (Fig. 2A). Similarly, CP70 cell viability was also suppressed with different concentration. At a concentration of 5 μM nobiletin cell viability was 96±1% (P<0.05), that was gradually inhibited to 26±3% by a 160-μM nobiletin treatment (P<0.01) (Fig. 2B). We also examined the growth inhibitory activity of nobiletin on IOSE-364 normal ovarian cells (Fig. 2C). We observed that nobiletin had a lower growth-inhibitory activity in IOSE-364 cells than in A2780 ovarian cancer cells. Therefore, our data suggest that nobiletin is somewhat more selective towards ovarian cancer cells than normal cells.

### Effect of nobiletin on ovarian cancer cell apoptosis

We ascertained that the growth inhibitory effects of nobeletin were due to cellular apoptosis. Annexin FITC assays revealed that nobiletin induced apoptosis in CP70 ovarian cancer cells in a concentration-dependent manner (Fig. 2D). CP70 cell apoptosis was 13±1% when it was not treated with nobiletin, which was significant increased at a concentration of 10 μM nobiletin (26±2%). CP70 cell apoptosis was gradually increased to 88±2% by a 160-μM nobiletin treatment (P<0.01).

### Effect of nobiletin on angiogenesis and growth of the tumor

**Figure 3:**
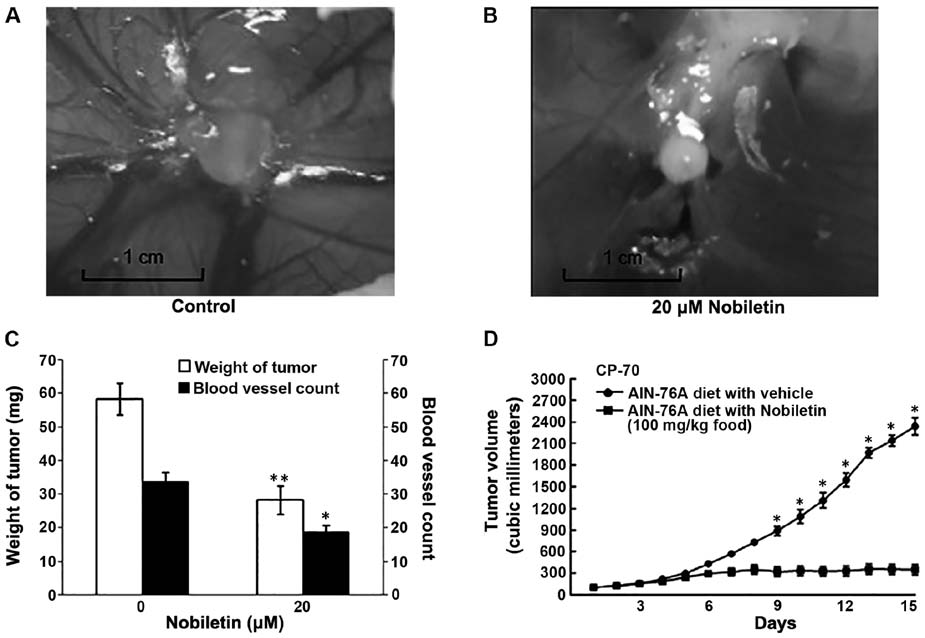
Nobiletin inhibits tumor growth and angiogenesis in CAM and athymic mouse models. Chicken chorioallantoic membrane (CAM) assay showing tumor without nobiletin treatment (A), treated with 20 μM nobiletin (B). CAM assays showed that 20 μM nobiletin suppressed the growth of blood vessels and A2780/CP70 tumors xenografted on chicken CAM relative to vehicle treated tumors (C). After 5 days of treatment, the tumors were excised, and the blood vessels were counted by phase contrast microscopy. Angiogenesis was evaluated by normalizing number of branching vessels to that of control CAM. The data are presented as mean ± SEM (n=10 eggs/group) (C). Nobiletin treatment also significantly inhibited tumor growth in the athymic mice (D).

To assess whether nobiletin inhibits the growth of human ovarian cancers *in vivo*, we used the CAM and athymic mouse models to study the effect of nobiletin on the growth rate of human ovarian cancer cells *in vivo*. The administration of 20 μM nobiletin significantly attenuated (P<0.01) the growth of OVCAR human ovarian tumors implanted on CAM (Fig. 3A and B). We counted the number of blood vessels to assess whether nobiletin was suppressing the growth of ovarian tumors by inhibition of angiogenesis. The chicken CAM experiment was repeated using A2780 cells and similar results were obtained. We observed that the treatment of OVCAR and A2780 cells with 20 μM nobiletin was able to inhibit angiogenesis. The implanted cancer cells grow to a tumor weight of 58±5 mg, with 29±4 blood vessels counted. Inclusion of 20 μM nobiletin in this implant, however, reduced tumor growth down to 34±3 mg (P<0.01) and inhibited blood vessel development to 19±2 (P<0.05). We speculated that the effect of nobiletin on the promotion of cancer cell apoptosis and inhibition of angiogesis lead to the growth attenuation of tumors implanted on CAM. A typical image (Fig. 3C) is shown to contrast the tumors with or without nobiletin in terms of both tumor size and angiogenesis. The great effect of nobiletin on inhibiting tumor growth was conformed *in vivo* using an athymic mouse model, where they exhibited smaller tumor size (Fig. 3D). The tumors implanted on mice grow to a volume of 2450 mm^3^ at the 15th day. However, the treatment of nobiletin (100 mg/kg food) inhibited tumor growth to 300 mm^3^.

### Effect of nobiletin on VEGF expression

**Figure 4:**
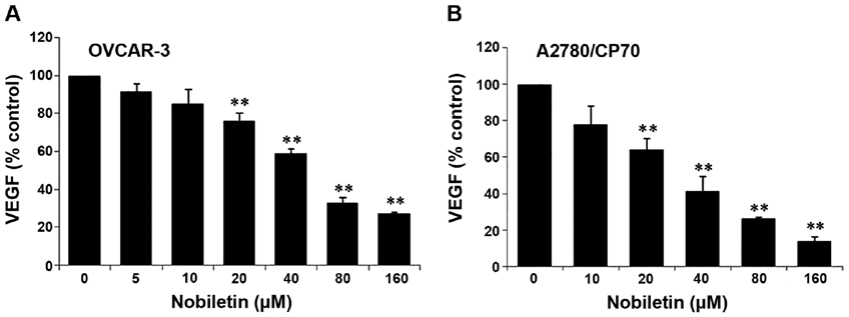
Nobiletin induces a concentration-dependent decrease of VEGF production from human ovarian cancer cells. The human ovarian cancer cell line OVCAR-3 (A), and A2780/CP70 (B) were treated with varying concentrations of nobiletin for 16 h. Subsequently, the VEGF levels in the supernatant was measured by ELISA. A total of three independent experiments, each in triplicates, were assayed, and the mean VEGF protein level from each duplicate was used for statistical analysis. ^*^P<0.05 as compared to control. ^**^P<0.01 as compared to control.

Our results showed that nobiletin significantly inhibited the expression of vascular endothelial grow factor (VEGF) in ovarian cancer cells, and this inhibition effect was enhanced with the increase of nobiletin concentration (Fig. 4). In both OVCAR-3 and A2780/CP-70 cells, the inhibitory effect of nobiletin on VEGF secretion reached a significant level when its concentration was >20 μM. The levels of secreted VEGF protein in OVCAR-3 cell culture supernates were downregulated to 77±2% at a concentration of 10 μM nobiletin (P<0.01) and to 28±1% at a concentration of 160 μM nobiletin (P<0.01) (Fig. 4A). Similarly the levels of secreted VEGF protein in CP70 cells ranged from 66±2% (20 μM) to 16±2% (160 μM) with respect to different nobiletin concentration (Fig. 4B).

### Signaling pathways underline the growth inhibiting activity of nobiletin

**Figure 5:**
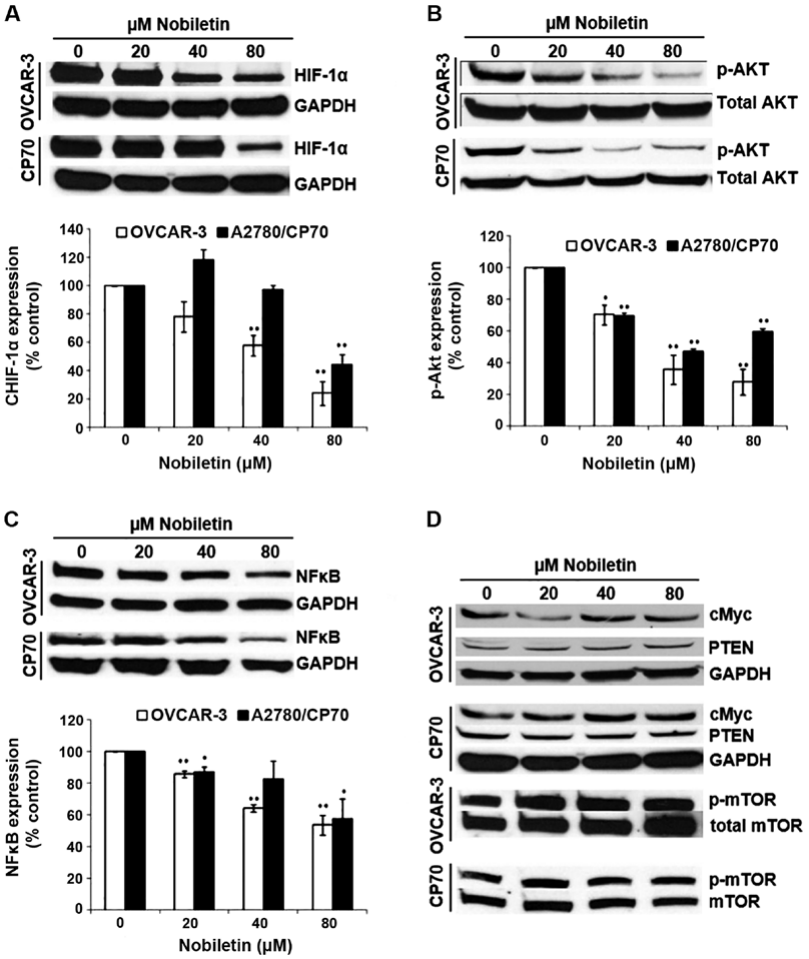
Nobiletin regulates several intracellular signaling pathways in human OVCAR-3 and CP-70 ovarian cells. (A) Nobiletin decreases the levels of HIF-1α in OVCAR-3 and CP-70 human ovarian cells. Protein bands were quantitated, normalized by corresponding GAPDH bands, and expressed as percentages of control (bar graph below the panel). (B) Nobiletin lowers the phosphorylation of Akt (Ser4) in OVCAR-3 and CP-70 cells. The levels of total Akt are not affected by nobiletin treatment. The bar graph below the panel represents densitometric analysis of the immunoblotting data. (C) Nobiletin decreases NF-κB expression in both ovarian cancer cell lines. (D) Nobiletin had no effect on expression of c-Myc, PTEN, mTOR proteins. Nobiletin had no effect on the phosphorylation of mTOR protein in either ovarian cancer cell line. A total of three independent experiments were carried out for statistical analysis. Data represent mean ± SE from 3 independent experiment. ^*^P<0.05 as compared to control. ^**^P<0.01 as compared to control.

HIF-1α is one of the key factors for the regulation of VEGF expression. As shown in Fig. 5A, nobiletin had a certain impact on HIF-1α expression of ovarian cancer cells. For OVCAR-3, 20-μM nobiletin treatment led to inhibition of HIF-1α protein to 78±6% by 24 h of treatment. Higher concentrations of nobiletin resulted in greater inhibition, with the levels of HIF1-α protein down to 57±3% by 40-μM nobiletin treatment (P<0.01) and 23±3% by 80-μM nobiletin treatment (P<0.01). However, for CP70, HIF-1α expression was slightly enhanced (118±2%) when the concentration of nobiletin was <20 μM and was significantly inhibited (47±2%) when its concentration was 80 μM (P<0.01). It seems CP70 cells were more resistant to the effect of nobiletin than OVCAR-3 cells on HIF-1α expression.

Nobiletin inhibited phosphorylation of AKT, which is known to be the major signal for cell survival and proliferation (33). As shown in Fig. 5B, nobiletin decreased AKT phosphorylation for both OVCAR-3 and CP70 ovarian cancer cells. P-AKT level was downregulated from 70±2% by 20-μM nobiletin treatment to 29±4% by 80-μM nobiletin treatment in OVCAR-3 cells. The phosphorylation of AKT was also inhibited from 69±1% by the 20-μM nobiletin treatment to 49±1% by 40-μM nobiletin treatment in CP70 cells. However, the effect of nobiletin on the inhibition of AKT phosphorylation in C70 cells was decreased when treated with 160-μM nobiletin. Again, it seems CP70 cells were more resistant to the effect of nobiletin than OVCAR-3 cells on AKT phosphorylation.

NF-κB is a common transcription factor that is related to many signal transduction pathways of cancer cell proliferation and angiogenesis (34,35). The effect of nobiletin on NF-κB expression is shown in Fig. 5C. Results showed that nobiletin had an impact on NF-κB (p50) expression. The level of NF-κB (p50) decreased with the increase of nobiletin concentration in OVCAR-3 and CP-70 cells. For OVCAR-3, the inhibitory effect reached a significant level (87±1%) when the concentration of nobiletin was 20 μM (P<0.01). In CP70, NF-κB (p50) level reduced with the increase of nobiletin concentration, and it reached a significant level when the nobiletin concentration was 20 (88±1%) and 80 μM (57±6%) (P<0.05).

c-Myc is a transcription factor that plays a role in cell cycle progression, apoptosis and cellular transformation. Unlike kaempferol, which inhibited c-Myc protein expression (35), our results indicated that nobiletin did not inhibit c-Myc expression in ovarian cancer cells (Fig. 5D). Its mechanism needs to be further investigated. PTEN acts as a tumor suppressor gene which is involved in the regulation of the cell cycle, preventing cells from growing and dividing too rapidly (36). The protein of mTOR is a serine/threonine protein kinase that regulates cell growth, cell proliferation, cell motility, cell survival, protein synthesis, and transcription (37). Results also showed that nobiletin had no significant effect on PTEN and p-mTOR expression in ovarian cancer OVCAR-3 and CP-70 (Fig. 5D).

### Nobiletin inhibits VEGF by regulating HIF-1α expression and Akt signaling

**Figure 6:**
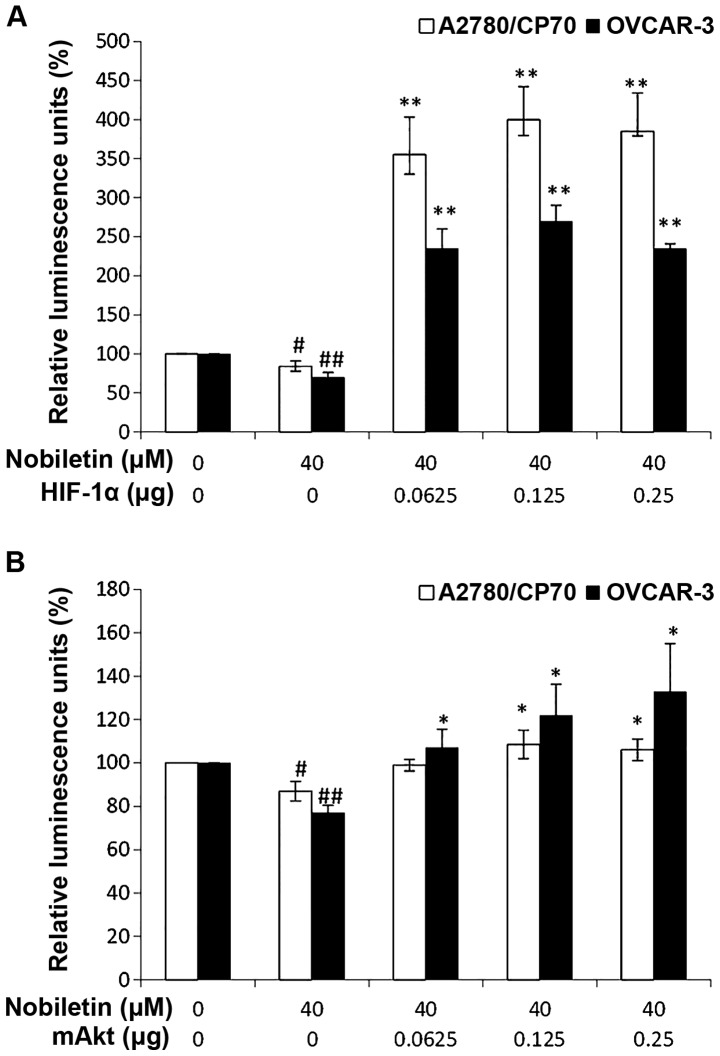
Forcing expression of HIF-1α protein (A) and Akt protein (B) reversed the effect of nobiletin on VEGF transcriptional activation. OVCAR-3 and A2780/CP70 cancer cells were seeded in a 96-well plate at 10,000 cells/well and incubated overnight. The cells were then transfected with 0.05 μg VEGF (Hif -1α) luciferase reporter, 0–0.25 μg HIF-1α (AKT) or SR-α plasmids by 0.6 μl jetPRIME reagent for 4 h, followed by 16-h treatment with 0 or 40 μM nobiletin. The cells were harvested and analyzed for luciferase and total protein levels, and the levels of VEGF (Hif -1α) reporter were normalized by corresponding total protein levels. Data represent mean ± SE from 3 independent experiment. ^*^P<0.05 as compared to control. ^**^P<0.01 as compared to control.

To see that HIF-1 is not only regulated by nobiletin treatment, but also plays a role in the nobiletin inhibition on VEGF expression, ovarian cancer cells were transfected with the VEGF-promoter reporter together with HIF-1 plasmids. While nobiletin treatment significantly inhibited VEGF transcriptional activation, this inhibition was concentration-dependent and significantly reversed by forced expression of HIF-1α protein (Fig. 6A).

The role played by Akt signaling in the nobiletin regulation of VEGF expression was investigated in both ovarian cell lines. It was found that the phosphorylation of Akt was significantly inhibited by 2-h nobiletin treatment (Fig. 6B). After transfecting with VEGF-promoter reporter and mAkt plasmids, VEGF transcriptional activation was significantly reduced by nobiletin treatment in both ovarian cancer cell types, and this effect was significantly reversed by forced expression of Akt protein (Fig. 6B).

## Discussion

Angiogenesis is a necessary condition for sustained tumor growth. Tumor cells take in nutrition and oxygen through the generated blood vessels, which produce substances needed for growth (38). Angiogenesis plays a central role in the development and progression of ovarian cancer (39). Histological studies demonstrated that ovarian tumors are richly vascularized. A correlation between microvascular count and biological aggressiveness was also found (40,41). Vascular endothelial growth factor (VEGF), as a key regulator of angiogenesis in ovarian cancer, is involved in various steps of ovarian carcinogenesis (42,43). VEGF plays a central role in tumor vasculature development and maintenance. VEGF expression promotes angiogenesis, thereby stimulating tumor growth and metastasis. Hypoxia-inducible factor 1α (HIF-1α) is a heterodimeric basic helix-loop-helix protein that directly activates transcription of VEGF gene by binding to a HRE (44). Previous studies showed that nobiletin inhibited angiogenesis in human umbilical vein endothelial cells (HUVECs) and zebrafish models (22,23,45). Similarly, the results showed that nobiletin effectively inhibited angiogenesis in ovarian cancer cells planted on chicken embryos models, indicating its potential to inhibit tumor growth *in vivo*.

While VEGF expression is an important factor in tumor growth and metastasis, HIF-1α is one of the key factors for VEGF expression. Previous studies (46,47) showed that the anticancer flavonoid compound kaempferol inhibited angiogenesis in ovarian cancer cells by downregulating HIF-1α expression. This is consistent with our study, indicating that the nobiletin inhibitory effects on angiogenesis can be traced back to suppression of HIF-1α expression. However, contradictory results were found in zebrafish models which showed that nobiletin increased mRNA levels of VEGF (22). This could be because nobiletin induced anti-angiogenesis through different mechanism in the two kinds of cells. PI3 kinase/Akt pathways could attenuate ovarian carcinoma through mediating angiogenesis and vascular permeability (48). In addition, taking into consideration previous studies that HIF-1α is related to the PI3 kinase/Akt signaling pathways (30–32), we tested if nobiletin inhibited expression of VEGF through PI3K/AKT pathways. Our results revealed that nobiletin significantly inhibited the phosphorylation of Akt. The inhibitory effect of nobiletin on Akt phosphorylation was more pronounced than on HIF-1α expression shown above. Previous studies showed that nobiletin suppressed PI3K/Akt pathways in human HepG2 (49). It was similar to our findings that nobiletin inhibited angiogenesis mainly through Akt pathways which results in the downregulation of VEGF expression (Fig. 6B). The mechanism by which nobiletin inhibits the PI3K/Akt signaling pathway is not fully understood. However, it has been shown that nobiletin suppressed invasion and migration of human gastric adenocarcinoma AGS cells through FAK/PI3K/Akt pathways (50).

**Figure 7:**
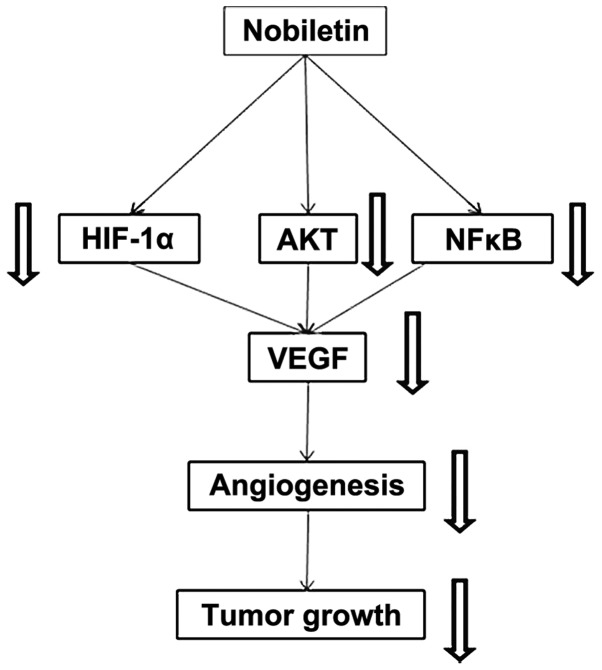
Proposed pathway for the nobiletin effect on tumor growth and angiogenesis.

Some researchers found that Akt pathways regulates the expression, activation and translocation of NF-κB (51–53). Suppression of NF-κB in tumor samples also inhibits proliferation, causes apoptosis, indicating the crucial role of NF-κB in cell proliferation and survival. We consider that it plays an important role on the nobiletin-induced apoptosis in A2780/CP70 cells. It was also found that Hif -1α promoter is responsive to selective NF-κB subunits, indicating that NF-κB is a direct modulator of Hif -1α expression (54). NF-κB, which is related to many signal transduction pathways of cancer cells (34), has been identified in tumors of epithelial origin such as breast, colon, lung and ovarian cancers (55). Recent research suggested the importance of NF-κB in the propagation of ovarian cancer cell lines (56). It was also found that NF-κB had a relationship with angiogenesis; that is, NF-κB regulates c-Myc expression. Overexpression of NF-κB removed the kaempferol inhibitory effect on c-Myc expression (35). Our results showed that the effect of nobiletin on NF-κB in ovarian cancer cells varies with cancer cells. Similar to the results found in AKT phosphorylation, NF-κB levels in the OVCAR-3 cells were more sensitive than those in the CP70 line. Nevertheless, nobiletin treatment significantly reduced NF-κB expression in both cell lines in a concentration-dependent manner. As previous study showed NF-κB have been linked to regulation of VEGF production (57), we conclude nobiletin antagonizes VEGF expression through NF-κB (Fig. 7).

The proposed mechanism by which nobiletin hampers angiogenesis involves lowering concentrations of VEGF regulators, namely HIF-1α and NF-κB (Fig. 7). We found nobiletin to have little effect on c-Myc, PTEN, and p-mTOR expression, which indicates that nobiletin does not inhibit expression of VEGF through PTEN/mTOR pathways, neither is c-Myc the key protein that is affected by nobiletin treatment in ovarian cells. Noteworthy, both HIF-1α and Akt were demonstrated to play direct roles in VEGF secretion. Overexpression of either protein in the presence of nobiletin neutralized its VEGF diminishing effects. Since Akt phosphorylation is intimately linked to HIF-1α activation, it is likely nobiletin exerts its cancer fighting properties through blocking Akt phosphorylation. Impeding Akt activity likewise reduces HIF-1α and NF-κB levels, subsequently dropping VEGF production and obstructing angiogenesis. Considering all the evidence, we believe this is the central pathway through which nobiletin mediates its tumor limiting effects.

Importantly, our *in vitro* research conducted agrees with both of our *in vivo* models. In our CAM model, nobiletin treatment significantly reduced not only the tumor size but also the number of blood vessels, confirming its potency in countering angiogenesis. Furthermore, nobiletin also suppressed tumor growth rates of CP-70 human ovarian cancer cells in the nude mouse model. The administration of nobiletin did not cause any discomfort in mice. The weights of mice, their food intake, water intake were unaffected by the administration of nobiletin (data not shown).

The challenge of conventional chemotherapy in ovarian cancer is that chemotherapeutic drugs are also toxic to normal ovarian epithelial cells. We find that nobiletin potently inhibits the viability of human ovarian cancer cells, and has minimal effects of the viability of normal ovarian cells. The selective inhibitory effect might be at least partly attributed to the apoptosis and anti-angiogenesis induced by nobiletin. Overexpression and activation of Akt, which results in the survival of cancer cells that normally undergo apoptosis (58), occurs in different kinds of cancers, such as gastric, lung, panacreatic and ovarian cancer (59). Nobiletin inhibits overian cancer cells selectively, possibly due to its effect on the phosphorylation of Akt.

Platinum drugs have been most frequently applied for the treatment of cancers including ovarian cancer. However, acquired resistance to conventional platinum based chemotherapy has become a major impediment in cancer treatment. Novel therapies that can reverse drug resistance or kill drug resistant ovarian cancer cells directly are highly desired. Flavonoid compounds like tangeretin (60) and kaempferol (61) have been shown to sensitize ovarian cancer cells to the apoptotic effects of cisplatin. Since nobiletin selectively inhibits ovarian cancer cells, it is expected that nobiletin may be useful both as a single agent as well as in combination therapies in the treatment of ovarian cancers.
